# Correction: How social capital helps communities weather the COVID-19 pandemic

**DOI:** 10.1371/journal.pone.0258021

**Published:** 2021-09-23

**Authors:** Christos A. Makridis, Cary Wu

Fig 2 is incorrect. The authors have provided a corrected version here.

**Fig 2:**
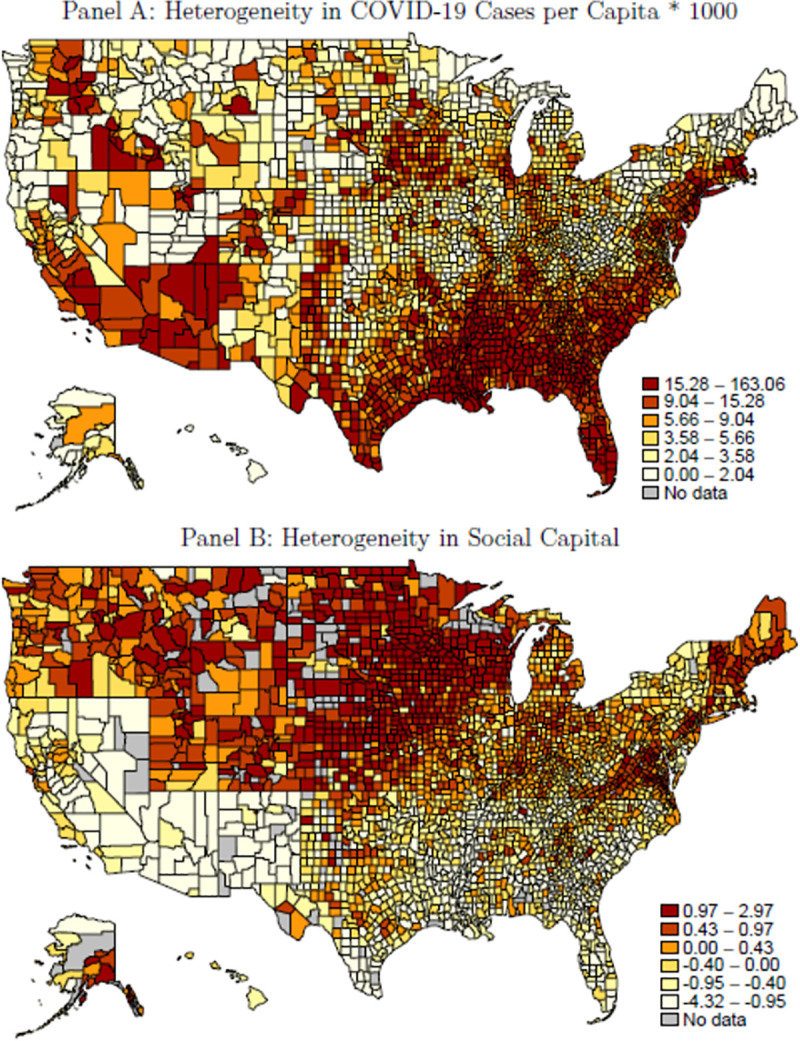
Spatial heterogeneity in infections per capita and social capital.
