# Supplementary material for: How social capital helps communities weather the COVID-19 pandemic
Source: PLoS One. 2021 Jan 29;16(1):e0245135. doi: 10.1371/journal.pone.0245135 (PMC7846018; doi:10.1371/journal.pone.0245135)
Supplement: Attachment — Submitted filename: Response to Reviewers.docx [file pone.0245135.s002.docx]

Dear Editor Freeman,

We are writing to resubmit our manuscript “*How Social Capital Helps Communities Weather the COVID-19 Pandemic*” (PONE-D-20-26570_R1) for reconsideration to *PLOS ONE.* To better reflect our main findings, we have changed our title to “*How Social Capital Helps Communities Weather the COVID-19 Pandemic?”* Thank you for your careful reading, and for the very fast review process concerning our manuscript.

We have made all the changes you suggested. Both authors have proofread the article and we hope it is now ready to be published.

Sincerely,

Authors

Review Comments to the Editor

1) p. 3 ``Although we recognize that these demographic controls are not exhaustive of all the omitted cross-sectional differences, we interpret the robustness of our estimates as suggestive evidence that additional bias would not qualitatively change our results. We nonetheless report conduct several robustness checks.”

The word choice of bias is odd here. Do you mean additional variables. Also, remove `report’ or ‘conduct’ from the last sentence.

You are right, thank you! We took out “additional” and “report.”

2) ``from Bailey et al. (2018) to construct a measure of the social capital that an individual in a county is exposed to social networks.

Something is not right here. Not clear what you mean be construct a measure of social capital that an individual in a county is exposed to social networks.

Good catch. The measure reflects the social capital in their social network. If an individual is connected with another county with high social capital, then that gets more weighted when constructing this measure.

3) ``Helliwell et al. (2014) also shows that societies with more social capital and trust can respond to economic crisis and institutional transitions more happily and more effectively.”

Check this; I think `shows’ should be `show’ If the subject is the authors’ article rather than the article, then it should be plural. Please check throughout the manuscript.

You are right. Whenever its et al, then it’s plural.

4) ``Furthermore, when societies have more trust and civic norms, they become less depend on formal institutions (Arrow 1972; Knowles 2007).” Dependent instead of depend?

Yes, exactly! Thank you.

5)``We also conduct robustness using the index of social capital from Chetty et al. (2014) at a commuting zone-level.”

The robustness of what to what? Your results to changes in how you estimate social capital? Please clarify.

We are testing the sensitivity to our measure of social capital. Just clarified in text.

6) Black and African American seem to be used interchangeably throughout. Pick one.

Thank you, we should be consistent!

7) ``In the U.S., social capital helps communities better able to respond to the COVID-19 pandemic through Americans’ greater commitment to achieving a common goal.”

`better able to’ should be something like ``higher social capital helps communities respond better than communities with lower social capital” the use of `better’ requires a comparison.

Thank you!

8) Figure 2. Please use an A and B to clarify which map displays social capital and infection data. Please clarify in the caption.

Thanks! We added that note to the file name in the upload.
